# The Function of Personality in Suicidal Ideation from the Perspective of the Interpersonal-Psychological Theory of Suicide

**DOI:** 10.3390/ijerph15040636

**Published:** 2018-03-30

**Authors:** Marc Baertschi, Alessandra Costanza, Alessandra Canuto, Kerstin Weber

**Affiliations:** 1 Service of General Psychiatry and Psychotherapy, Nant Foundation, Avenue des Alpes 66, 1820 Montreux, Switzerland; 2 Faculty of Psychology, University of Geneva, Boulevard du Pont d’Arve 40, 1205 Geneva, Switzerland; 3 Psychiatry Department, SS. Antonio e Biagio e Cesare Arrigo Hospital, Via Venezia 16, 15521 Alessandria, Italy; alessandra.costanza@ospedale.al.it; 4 Executive and General Management Service, Nant Foundation, 1804 Corsier-sur-Vevey, Switzerland; alessandra.canuto@nant.ch; 5 Division of Institutional Measures, Medical Direction, Geneva University Hospitals, Les Voirons—Chemin du Petit-Bel-Air 2, 1225 Chêne-Bourg, Switzerland; kerstin.weber@hcuge.ch

**Keywords:** personality, Five-Factor Model, Interpersonal-Psychological Theory of Suicide, suicidal ideation

## Abstract

The Interpersonal-Psychological Theory of Suicide (IPTS) has been increasingly studied over the last years, responding to the demand for a valid framework addressing suicidality. Yet, only a few studies have explored the function of personality in the IPTS and none with clinical patients. We aimed to contribute to fill this gap in investigating the relationship between personality as conceptualized by the Five-Factor Model, the IPTS constructs, and a dimensional measure of current suicidal ideation. We conducted correlation, multiple linear regression, and path analyses based on a trait-interpersonal framework in a sample of 201 individuals visiting the psychiatric emergency room of a general hospital with current suicidal ideation. Neuroticism (positively) and openness (negatively) predicted perceived burdensomeness, while neuroticism (positively) and extraversion (negatively) predicted thwarted belongingness. Higher conscientiousness and lower extraversion were both predictors of the acquired capability for suicide. However, none of the models involving path analyses with IPTS variables as mediators of the relationship between personality traits and suicidal ideation was adequately adjusted to the data. Thus, it appears that personality plays a significant albeit modest role in suicidality when considered from an IPTS perspective. As personality is frequently assessed in the clinical routine, health professionals should consider it as complementary to detect individuals at risk of or presenting suicidal ideation.

## 1. Introduction

The clinical management of patients who attempted suicide or have suicidal ideation suffers from the scarcity of comprehensive models accounting for the suicide phenomenon [1]. The Interpersonal-Psychological Theory of Suicide (IPTS) was designed in this regard as a framework proposing that the most dangerous kind of desire for suicide stems from two constructs, namely feelings of not belonging to one’s social group (thwarted belongingness) and the perception of being a burden for significant others (perceived burdensomeness). Additionally, the theory specifies that acting on this desire for suicide is only possible if one has acquired the capacity to do so, assessed by a third construct labeled the acquired capability for suicide [1,2].

Tested in an increasing number of studies during the past decade, the predictions of the IPTS have not been unanimously supported [3]. In this context, further research on possible determinants of thwarted belongingness (TB), perceived burdensomeness (PB) and the acquired capability for suicide (AC) is warranted. Specifically, it has been pointed out that the IPTS does not account for the *propensity* to engage in suicide behavior. This, in other words, refers to possible pre-existing vulnerabilities for suicide, which could notably be appraised in terms of personality [4]. To our knowledge, the founding texts of the IPTS did not mention personality, although its role has been considered in recent IPTS studies from both categorical [5,6] and dimensional perspectives [7,8,9,10,11,12,13,14].

A trait model of personality, the Five-Factor Model (FFM) [15] has been adopted as consensual framework to determine each person’s individuality as a function of five broad dimensions: neuroticism (N), extraversion (E), openness (O), agreeableness (A) and conscientiousness (C). These dimensions have been progressively identified in the second half of the twentieth century starting with the pioneering work of Eysenck [16] who initially identified the “Big Two” N-E pair. Neuroticism refers to a temperamental measure of emotional health comprising components such as fear, anger and impulsivity. Extraversion relates to variables like warmth, assertiveness and activity, and has been viewed as a predisposition toward positive effects. Taken together, N and E constitute independent, nonetheless interrelated, sources of subjective well-being [17]. The “high N/low E” pattern is notably predictive of depressive symptoms and hopelessness [18] as well as suicidal ideation [19]. Agreeableness describes traits usually displayed in interpersonal relationships, such as courtesy, altruism, and compliance. Openness has often been associated with intellect and depicts a tendency toward a variety of experiences, ranging from inner thoughts or fantasy to actions and ideas (hence frequently referred to as “openness to experience”). Finally, conscientiousness concerns variables such as self-discipline, deliberation and dutifulness, and has been associated with educational achievement and volitional aspects.

Despite the absence of theoretical assumptions, the existing literature allows us to make inferences on the way personality traits could account for variability in IPTS constructs. Derived from the FFM, the Five-Factor Theory of personality [20] postulates that traits “influence patterns of thoughts, feelings, and actions” (p. 165). It could therefore be hypothesized that an individual’s expression of PB, TB and AC may partly stem from the specificity of his/her personality traits.

First, PB and TB have a strong cognitive component as they develop from non-necessarily objective *perceptions* of lack of connectedness or social isolation [2,21]. High levels of N have been associated with distorted cognition [22] and high levels of E with perceived social support, notably belongingness [23]. Additionally, high levels of A have been related to the ability of maintaining positive interpersonal relationships and to interpersonal adjustment [24]. A tentative hypothesis would thus be that a conjoint presence of high N, low E and low A creates favorable dispositions for PB and TB.

The only five studies quantitatively addressing the relationships between the FFM and the IPTS [4,9,11,13,14] support this assumption. Indeed, N was positively associated with PB, and E negatively associated with both PB and TB [9,13]. N was also positively and E negatively associated with suicide proneness [11,14], a construct including “engagement in overtly suicidal behavior as well as in risk-taking and potentially injury-producing behaviors, coupled with a lack of health and safety behaviors, and/or a lack of self-worth and self-enhancing behaviors” ([25], p. 416). Additionally, Cramer and colleagues [11,13,14] tested the mediating effects of IPTS variables on the relationship between the FFM and suicide proneness using structural equation modelling (SEM). Consistently over three different populations of pre-incarcerated offenders, members of the lesbian, gay and bisexual community, and university students, they found that, respectively, PB positively mediates the influence of N and TB negatively mediates the influence of E on suicide proneness. Moreover, the two studies including O, A and C in the SEM analyses [11,14] showed that TB negatively mediates the influence of A on suicide proneness, and that neither PB nor TB have a mediating effect on the pathway between O and suicide proneness. Other effects were also identified but inconsistently, which led the authors to suggest that trait-interpersonal pathways articulate differently in various population groups [11]. The same hypothesis was raised by Ireland and York [4], whose sample was comprised of women prisoners, to explain the unexpected association between increased extraversion and self-injurious behavior.

Second, the IPTS proposes that AC is progressively acquired through pain and fear exposure via mechanisms of habituation and opponent processes [2]. The question arises as to whether certain patterns of personality traits could favor the activation of these mechanisms. Habituation has been traditionally defined as a “decrement in magnitude of unlearned responses (…) due to repetitive stimulatory activation” ([26], p. 385). It has been experimentally assessed through a variety of physiological measures such as the acoustic startle response and skin conductance recording. To our knowledge, the relationship between habituation and personality has never been specifically studied within the context of AC; however, should a certain pattern of personality traits foster habituation in general, this could also apply to fear and pain habituation in particular, the latter two dimension being germane to AC. We only found one study addressing the relationship between personality, pain and habituation [27], and this study showed that individuals with low N habituate faster to pain than those with high N levels. However, in another study using the FFM, individuals with a personality profile of high N/low E-O-A-C displayed smaller amplitude of the P300 component of the auditory event–related cortical potential, which suggests faster habituation [28]. Although less directly relevant to AC, habituation and personality in general have not yielded constant associations, as illustrated by studies using an acoustic startle response paradigm. Faster habituation was associated with high-N individuals by Blanch and collaborators [29] but with high-E participants by LaRowe and colleagues [30], while Akdag et al. [31] did not find any relation with personality traits at all. Differences in theoretical approaches and measurement methods have probably fostered such inconsistency. In comparison with N and E, the other three FFM dimensions have been far less subject of investigations.

For these reasons, research on personality and the IPTS mainly remains exploratory at the current stage. This study was therefore designed to add a further contribution to the existing literature in; first, investigating the predictive character of FFM personality traits on the three main constructs of the IPTS and a measure of suicidal ideation, and; second, assessing the effects of IPTS variables as mediators of the relationship between personality traits and suicidal ideation. Based on the existing data, we expected that N would positively predict PB and E negatively predict PB and TB. We also hypothesized that PB would mediate the positive influence of N, and TB the negative influence of both E and A, on suicidal ideation. This boils down to replicate the hypothesized trait-interpersonal pathways progressively elaborated by Cramer and collaborators [11,13,14]. Additionally, we decided to investigate the possible mediating function of AC in the relationship between FFM traits and suicidal ideation. Although the IPTS posits that AC is independent from suicide desire [2], a relationship between these two constructs was found in about 57% of reviewed studies [3]. Because of the absence of consistent findings in the habituation literature, we included the five FFM personality traits as predictors in our analyses. 

## 2. Materials and Methods

### 2.1. Participants and Procedure

The study procedure was made of self- and clinician-administered questionnaires including a range of sociodemographic inquiries. A total of 368 individuals visiting the psychiatric room of the emergency department at the Geneva University Hospitals, Switzerland, for a suicide attempt or suicidal ideation were offered to participate to the study. Among them, 129 refused for various reasons such as difficulty concentrating, lack of interest, and problems understanding French. Additionally, 38 were excluded by the psychiatrist in charge of study inclusion due to serious suspicion regarding data quality after noticing obvious lack of motivation—illustrated by repeated patterns of answers or systematic refusal to fill in questionnaires—or procedural problems (e.g., absence of signed consent form). Thus, the final sample was comprised of 201 participants, aged 33.4 ± 14.5 years old, predominately composed of women (60.7%), Swiss citizens (57.7%), currently not in a relationship (65.7%), without children (60.2%), and either working or studying (57.7%). Most participants had a history of suicide attempts (63.2%) and a current psychiatric diagnosis (88.6%) according to the Mini-International Neuropsychiatric Interview [32] with the most prevalent diagnosis being an episode of major depression (69.7%), followed by alcohol dependence (16.9%) and non-alcohol substance dependence (10%). Additional sociodemographic information on the sample and recruitment procedure has been published elsewhere [33]. The study procedure and contents were approved by the local Research Ethics Committee and all participants signed an informed consent before study entry.

### 2.2. Measures

The personality profile was evaluated with the French version of the revised NEO-Five-Factor Inventory (NEO-FFI-R) [34,35], whose 60 items were selected from the longer NEO PI-R [36] in order to provide a score for the five personality dimensions of the FFM (i.e., N, E, O, A and C). Each item is calculated with a 0–4 Likert score, leading to a maximum score of 48 per dimension. Internal consistencies were good for C (α = 0.849, 95% CI (0.816, 0.878)), acceptable for N (α = 0.758, 95% CI (0.705, 0.805)) and E (α = 0.705, 95% CI (0.641, 0.762)), and questionable for O (α = 0.681, 95% CI (0.612, 0.741)) and A (α = 0.680, 95% CI (0.611, 0.742)).

The desire for suicide, as conceptualized by PB and TB, was assessed with the revised version of the Interpersonal Needs Questionnaire (INQ-R) [37], which proposes a subscale to evaluate PB (6 items, score range: 6–42) and a subscale to evaluate TB (9 items, score range: 9–63). For both scales, higher scores indicate stronger feelings of the construct measured. Internal consistency was good for PB (α = 0.866, 95% CI (0.835, 0.893)) and acceptable for TB (α = 0.749, 95% CI (0.693, 0.798)).

The acquired capability for suicide was assessed with the German Capability for Suicide Questionnaire (GCSQ) [38], which proposes three subscales of fearlessness of death (5 items, score range: 5–25), pain tolerance (five items, score range: 5–25), and perceived capability for suicide (1 item, score range: 1–5). The summation of these subscores provides a total score for AC. In all subscales, higher scores indicate greater capability for suicide. The internal consistency of the GCSQ was good (α = 0.880, 95% CI (0.854, 0.903)).

We finally assessed suicidal ideation with the Scale for Suicide Ideation (SSI) [39], a self-administered questionnaire comprised of 19 items rated on a 3-point Likert scale (score range: 0–38). The SSI has been validated in French [40,41]. In our sample, internal consistency was good (α = 0.844, 95% CI (0.811, 0.874)). In the absence of versions validated in French, the GCSQ and the INQ-R were translated and adapted according to the WHO guidelines [42] (http://www.who.int/substance-abuse/research_tools/ translation/en/).

### 2.3. Statistical Analyses

A full set of descriptive statistical analyses including means, standard deviations, skewness, kurtosis, and bivariate Pearson’s correlations were initially computed for all variables of interest. We then explored the predictive value of the FFM personality traits (N, E, A, O, and C) in the variance of each IPTS variable (PB, TB, and AC) as well as of a measure of current suicidal ideation (SI) with multiple linear regression models. Missing data appeared in three cases out of 201, which constitutes a ratio of 0.015, and were handled with multiple imputation. In line with the recommendations of White, Royston and Wood [43], who suggested that the imputation number should be equal or superior to the percentage of cases containing missing values, we conducted statistical analyses after generating five datasets. We decided to rely on original data—which included missing data—to assess parameters that could not be computed through combined estimation from multiple imputations (e.g., standard deviations, Fisher’s test in multiple regression models). These analyses were performed with SPSS version 24.0 (IBM SPSS, Chicago, IL, USA). Exploratory and control analyses (e.g., residuals, outliers, heteroscedasticity, multicolinearity) were conducted for every analysis. No assumption violation was observed.

In a second step, we investigated the mediating function of IPTS variables in the relationship between FFM personality traits and current SI using a path analysis framework. To this end, we specified six models. The first one drew on the literature and hypothesized that PB mediates a positive relationship between N and SI and a negative relationship between E and SI, whereas TB mediates negative relationships between E and SI and between A and SI. We then compared this model with a nested model adding direct pathways between FFM variables (i.e., N, E, and A) and SI. A similar procedure was replicated for the next two models. Both explored the mediating function of AC in the relationship between the FFM and SI, the second one including direct effects; yet, in light of the sparse, inconsistent findings addressing personality and AC (or rather AC’s possible proxies) we decided to include the five FFM variables as predictors without further specifying research hypotheses.

Finally, we tested two additional models that included PB/TB, respectively AC, as mediating variables. These models were designed in including FFM, IPTS and SI variables showing significant relationships in regression analyses. We expected these models to demonstrate a better fit to the data than the above-mentioned models based on the literature; however, direct statistical comparisons were not possible as the last two models were not nested within the initial four.

Path analyses were conducted using Amos version 23.0 (IBM SPSS, Chicago, IL, USA), which estimates missing data with a full information maximum likelihood estimator. Amos nevertheless does not compute confidence intervals for parameters estimated from databases containing missing data, as well as standard errors and *p*-values for some analyses (e.g., indirect effects). We used Statistica version 12.7 (StatsSoft Inc., Tulsa, OK, USA) to estimate the *χ*^2^ difference between nested models (i.e., model 1 vs. model 2, and model 3 vs. model 4).

## 3. Results

### 3.1. Descriptive Statistics

Means, standard deviations, skewness, kurtosis, and bivariate Pearson’s correlations for the variables of interest of this study are displayed in Table 1. Taking sample size into account, *Z*-test values for skewness (between |0.052| and |2.488|) and kurtosis (between |0.064| and |2.825|) suggest that normality was preserved in the sample distribution of each variable [44]. The four personality traits of E, O, A, and C were all positively associated with one another, except for O and C whose correlation was not significant. On the other hand, N was negatively related to E and C, and positively to O. The IPTS variables of PB and TB were positively related to each other and to SI. The variables associated with AC were SI, PB and C, all positively. Other IPTS-FFM significant relationships were N with PB and TB (both positively), E with PB and TB (both negatively), and A with TB (negatively). Finally, significant correlations between FFM traits and SI concerned N (positively), E and A (both negatively). Ranging from |0.143| to |0.453|, significant correlations represented small to medium effect sizes (*r*) according to the criteria of Cohen [45]. 

### 3.2. Regression Analyses

We first built three multiple linear regression equations including the five personality dimensions as predictors and, respectively, PB, TB, and AC as the dependent variable. The initial model accounted for a significant proportion of the variance in PB, *F*(5, 193) = 5.115, Adjusted *R^2^* = 0.094, *p* < 0.001. The criterion was predicted by increased levels of N (*b* = 0.422, *t* = 4.186, *p* < 0.001) and decreased levels of O (*b* = −0.251, *t* = −2.248, *p* = 0.025). However, no regression effect was found for E (*b* = −0.160, *t* = −1.391, *p* = 0.164), A (*b* = −0.024, *t* = −0.212, *p* = 0.832), and C (*b* = 0.048, *t* = 0.563, *p* = 0.573). The second model explained a significant proportion of the variance in TB, *F*(5, 193) = 7.175, Adjusted *R^2^* = 0.135, *p* < 0.001, with TB being predicted by N (*b* = 0.338, *t* = 3.265, *p* = 0.001) and E (*b* = −0.364, *t* = −3.092, *p* = 0.002). While the predicting value of O (*b* = −0.223, *t* = −1.932, *p* = 0.053) and A (*b* = −0.213, *t* = −1.868, *p* = 0.062) approached the significance threshold, C (*b* = 0.084, *t* = 0.954, *p* = 0.340) was not related to the criterion. Finally, the set of FFM variables predicted variance in AC, *F*(5, 193) = 3.203, Adjusted *R^2^* = 0.053, *p* = 0.008, with specific effects found in E (*b* = −0.185, *t* = −2.372, *p* = 0.018) and C (*b* = 0.191, *t* = 3.327, *p* = 0.001) but not in N (*b* = −0.070, *t* = −1.032, *p* = 0.302), O (*b* = 0.026, *t* = 0.347, *p* = 0.729), and A (*b* = −0.100, *t* = −1.332, *p* = 0.183).

We then constructed a fourth model with SI measured by the SSI as the dependent variable and the similar five personality variables as predictors. Additionally, we controlled for two socio-demographic variables susceptible to have a confounding influence, namely gender and age. Suicidal ideation has indeed been identified as more frequent in female than male gender [46,47], and tends to be more prevalent in older than younger age groups [48,49]. We transformed gender into a dummy variable, coding 0 for females and 1 for males. The model predicted SI, *F*(7, 192) = 7.877, Adjusted *R^2^* = 0.195, *p* < 0.001, as well as higher N (*b* = 0.304, *t* = 3.743, *p* < 0.001) and lower E (*b* = −0.416, *t* = −4.598, *p* < 0.001). However, no effect was found for O (*b* = −0.062, *t* = −734, *p* = 0.463), A (*b* = −0.141, *t* = −1.662, *p* = 0.097) and C (*b* = 0.100, *t* = 1.535, *p* = 0.125). Similarly, neither age (*b* = −0.017, *t* = −0.423, *p* = 0.672) nor gender (*b* = −1.232, *t* = −1.096, *p* = 0.273) had a significant influence on SI. 

### 3.3. Path Analyses

As detailed in Table 2, path analyses with PB and TB as mediators were poorly adjusted to the data. This was the case for models without (*χ*^2^ = 65.241, df = 9, *p* < 0.001, CFI = 0.609, RMSEA = 0.177 90% CI (0.138–0.219)) and with (*χ*^2^ = 42.551, df = 6, *p* < 0.001, CFI = 0.747, RMSEA = 0.175 90% CI (0.127–0.226)) direct estimates of FFM traits on SI. Although model 2 yielded three degrees of freedom to model 1, the former was significantly improved (Δ*χ*^2^ = 22.69, Δdf = 3, *p* < 0.001). All direct effects were significant except A to SI in model 2. Despite *p*-values were unavailable for PB and TB mediation effects of FFM traits on SI, standardized effects ranged from |0.021| to |0.115| suggesting that these effects are weak.

Next we tested a model (model 5) based on significant predictive relationships highlighted in regression analyses. Thus, we included N, E, and O in the model and tested the following direct effects: N to PB, N to TB, N to SI; E to TB, E to SI; O to PB. As displayed in Table 4, all direct effects were significant but the model did not adjust well to the data (*χ*^2^ = 60.073, df = 7, *p* < 0.001, CFI = 0.681, RMSEA = 0.195 90% CI (0.151–0.242)).

In the same vein, path analysis frameworks using AC as mediator of the effect of FFM traits on SI did not demonstrate adequate fit to the data in both models (model 3: *χ*^2^ = 142.311, df = 15, *p* < 0.001, CFI = 0.111, RMSEA = 0.206 90% CI (0.176–0.238); model 4: *χ*^2^ = 92.562, df = 10, *p* < 0.001, CFI = 0.423, RMSEA = 0.203 90% CI (0.166–0.242)) as described in Table 3. Despite the loss of five degrees of freedom, model 4 had a significantly better adjustment than model 3 as assessed by the Chi-squared analysis (Δ*χ*^2^ = 49.749, Δdf = 5, *p* < 0.001). In both models, E (negatively) and C (positively) had an influence on AC, and AC positively predicted SI. Additionally, direct effects of N (positively) and E (negatively) on SI were found in model 4. Standardized indirect effects of FFM traits on SI showed low values ranging from |0.006| to |0.064|. 

Next, we tested a model (model 6) with AC playing a mediating role between FFM variables and SI based on the regression analyses. We included N, E and C, and estimated the following relations: N to SI; E to AC, E to SI; C to AC. As displayed in Table 4, all direct effects were significant but the model did not adjust well to the data (*χ*^2^ = 31.452, df = 5, *p* < 0.001, CFI = 0.705, RMSEA = 0.163 90% CI (0.111–0.219)).

## 4. Discussion

This study is the first to investigate the relationship between personality dimensions as defined by the FFM model and the main constructs of the IPTS in a large clinical population with current SI. Our results from correlation and regression analyses are globally in line with the literature and suggest that certain dispositions of personality traits may foster the occurrence of PB, TB and AC. When significant findings occurred their effect size remained globally modest, as illustrated by the percentages of variance in PB and TB (9.4% and 13.5%, respectively) explained by FFM traits through regression equations. However, we did not replicate the findings of Cramer et al. [11,13,14], notably with regard to the trait-interpersonal hypothesis. Overall, this suggests that personality does play a role in the variance of SI or variables approaching SI (that is, in this case, PB and TB from the IPTS) but that the importance of this role should be considered with caution and not yield to over-interpretation. Interestingly, personality was found to be predictive of AC as well. This is, to our knowledge, the first time that an association between personality and AC is statistically assessed.

In line with the literature and in both correlation and regression analyses, we found a relationship between N and PB, and between N, E and TB. Additionally, O was a negative predictor of PB, and E and A negatively correlated with PB, respectively TB. An association between N, E, and variables related to suicide desire has already been identified [9,50]. Additionally, a conjoint presence of higher levels of N and lower levels of E, A and C characterized individuals with psychiatric disorders [51] and more specifically SI [19]. Whereas C was associated with neither PB nor TB in our study, the correlation between A and TB was not surprising as agreeableness is strongly related to interpersonal behavior. Besides, low levels of A might complicate the formation of interpersonal relationships, indirectly fostering feelings of not belonging to a social group [36]. Similarly, the finding that PB was negatively predicted by O should not be a surprise when one considers that “openness affects social perceptions and the formation of social attitudes, the choice of friends and spouses, political activity, and cultural innovation” ([52], p. 257). Thus, our results suggest that the way one considers interpersonal relationships is pivotal in the formation of SI, providing in this way support to the IPTS.

High levels of C and low levels of E were predictive of a greater capability to act on one’s suicidal desire, which implies that personality can directly or indirectly contribute to the way one’s may acquire the capability to commit suicide as suggested by others [7,8]. These studies yet addressed dimensions not considered by the FFM (e.g., psychotism, stoicism, sensation seeking), which limits comparisons with ours. The fact that E played a role in the acquisition of a construct such as AC, which is comprised of a habituation component, is in line with other studies that found a relationship between E and habituation. However, in contrast with our results, these studies often concluded that habituation takes place faster in extroverts than introverts [30,53,54]. From another standpoint, it has been underscored that medically serious suicide attempters—that is, individuals supposed to have acquired the capability to seriously harm themselves—thought more about suicide preparation and were more precautious against the discovery of their intentions than individuals whose suicide attempts were medically less serious [55,56]. This implies that those with medically serious suicide attempt showed features corresponding to high scores on the C dimension such as achievement striving, self-discipline and deliberation. As participants in our study were included with a mixed criterion of presence of SI and presence of a suicide attempt, as well as different histories of suicide attempts, we were not able to investigate this aspect more specifically. Thus, further research should be conducted, on the one hand, to confirm the role of E in the development of habituation and other components from which AC theoretically derives (e.g., opponent processes), and, on the other hand, to specify the interplay of personality and AC in taking different levels of lethality into consideration.

We could not replicate the findings of Cramer and colleagues [11,13,14] in that none of the trait-interpersonal model tested, either with PB/TB or with AC, fit the data in an adequate way. This was the case for theory-driven models (i.e., models 1 to 4) and models based on regression findings (i.e., models 5 and 6). This is somewhat surprising as direct effects between FFM, IPTS and SI variables were consistently significant; yet the limited sample size and the relative small proportion of variance in IPTS components and SI explained by regression models might imply a lack of statistical power that could account for the poor fit of global models. Similarly, these results might be due to the type of population studied (i.e., patients visiting emergency with suicidal ideation) as it has been proposed that relationships between personality and suicidality could depend on this [4,11]. Another possible reason, our measure of outcome was SI while that of Cramer and colleagues was suicide proneness. Yet, suicide proneness has been associated with SI [25], making this assumption unlikely. Finally, contrary to Cramer and collaborators [11,13], we did not allow measurement errors of some of our variables to correlate. While such a procedure may lead to significant improvement of a path analysis model, it appears to be theoretically unjustified in almost all cases [57]. Cramer and colleagues did not provide a detailed rationale for allowing measurement errors to correlate and, as far as we were concerned, we did not have any. It appears then pivotal that trait-interpersonal models continue to be investigated under theory- and evidence-based hypotheses, with various population types and important sample sizes. This constitutes a prerequisite before any definitive conclusions on the viability of this approach can be drawn.

This study had several limitations, which should be taken into account. First, our experimental design did not allow us to collect longitudinal data, which limits the interpretation of the findings in a predictive way. Second, we defined our research hypotheses on the basis of the existing literature. However, studies exploring the relationships between suicide behaviors, the FFM and the IPTS did not consider the same measurement outcomes. Thus, while we used a measurement of SI, others utilized history of SI [9], suicide proneness [11,13,14], and self-injurious behavior [4]. These major differences may have contributed to some of the inconsistent results observed. Third, low internal consistency values in two subscales of the NEO FFI-R, namely O and A, might imply that items were not sufficient in number to satisfactorily account for these dimensions. This may have impacted our results. In line with that, the use of the 60-item NEO FFI-R rather than the longer, 240-item NEO PI-R did not offer the opportunity to analyze the facets of the FFM dimensions. Future studies are encouraged to utilize the NEO PI-R as it proposes a personality profile based on 30 facets, although administration might be difficult to implement in the clinical practice of patients with active suicidal behavior. 

## 5. Conclusions

The clinical use of the FFM through specific instruments such as the NEO PI-R or the NEO-FFI-R provides health professionals with information potentially useful in their daily practice. It allows them to identify directions in which the treatment could evolve and to construct strong therapeutic alliances, offering patients and significant others opportunities to become aware of elements remained unconscious, or suggesting patient-specific therapeutic frameworks [58]. However, and despite that clinical research has underscored the relevance of the FFM in psychotherapy [59,60,61], the reliability of these instruments in the detection of individuals at risk of suicidal events is yet to be supported by additional research.

Bearing in mind that other factors may play a more important part of variance in suicidality than personality, this paper advocates personality as a potential determinant of the desire for suicide and the acquired capability for suicide as conceptualized by the IPTS, a leading theoretical framework in suicidality. Thus, the administration of a personality inventory in the clinical routine should be encouraged, as it could provide health professionals with relevant information regarding the risk of a patient to develop future suicidal ideation or engage in suicidal behavior.

## Figures and Tables

**Table 1:** Means, standard deviations and bivariate Pearson’s correlation matrix for variables of interest (*n* = 201 with five imputations).

Variables	Mean	SD †	Skewness †	Kurtosis †	1	2	3	4	5	6	7	8	9
Neuroticism	33.703	7.470	−0.428	−0.357	1								
Extraversion	25.185	6.434	−0.340	0.047	−0.176 *	1							
Openness to experience	28.678	6.699	0.097	−0.235	0.269 **	0.187 **	1						
Agreeableness	30.735	6.656	−0.413	−0.022	−0.086	0.191 **	0.175*	1					
Conscientiousness	26.967	8.990	−0.185	−0.306	−0.217 **	0.296 **	0.118	0.342 **	1				
Perceived burdensomeness	23.375	10.120	0.009	−0.966	0.283**	−0.179 *	−0.083	−0.081	−0.088	1			
Thwarted belongingness	40.020	10.661	−0.164	−0.304	0.233 **	−0.292 **	−0.132	−0.198 **	−0.109	0.380 **	1		
Acquired capability for suicide	29.898	6.696	0.149	−0.100	−0.089	−0.103	−0.019	−0.034	0.189 **	0.143 *	0.100	1	
Suicidal ideation	14.707	8.134	−0.268	−0.779	0.322 **	−0.358 **	−0.038	−0.167 *	−0.099	0.453 **	0.369 **	0.251 **	1

Note: * *p*-values significant at the 0.05 threshold; ** *p*-values significant at the 0.01 threshold; † computed with the original data, i.e., *n* = 200 (except for suicidal ideation, *n* = 201). The standard error was 0.172 for skewness and 0.342 for kurtosis.

**Table 2:** Estimated unstandardized (with standard errors) and standardized parameters for the regression weights, as well as fit indices, for the two models with PB and TB as mediators in the relationship between FFM variables and SI.

**Model 1, fit indices: *χ*^2^ = 65.241, df = 9, *p* < 0.001, CFI = 0.609, RMSEA = 0.177 90% CI (0.138–0.219)**			
Parameter estimates	Unstandardized *β*	Standardized *β*	*p*-value
N to PB	0.345 (0.091)	0.256	**<0.001**
E to PB	−0.221 (0.106)	−0.141	**0.038**
E to TB	−0.437 (0.111)	−0.266	**<0.001**
A to TB	−0.237 (0.108)	−0.149	**0.027**
PB to SI	0.294 (0.050)	0.375	**<0.001**
TB to SI	0.175 (0.047)	0.235	**<0.001**
N to SI (indirect)	0.101	0.096	N/A
E to SI (indirect)	−0.141	−0.115	N/A
A to SI (indirect)	−0.041	−0.035	N/A
**Model 2, fit indices: *χ*^2^ = 42.551, df = 6, *p* < 0.001, CFI = 0.747, RMSEA = 0.175 90% CI (0.127–0.226)**			
Parameter estimates	Unstandardized *β*	Standardized *β*	*p*-value
N to PB	0.342 (0.092)	0.254	**<0.001**
E to PB	−0.215 (0.106)	−0.137	**0.043**
E to TB	−0.437 (0.111)	−0.266	**<0.001**
A to TB	−0.238 (0.108)	−0.150	**0.027**
PB to SI	0.250 (0.049)	0.321	**<0.001**
TB to SI	0.104 (0.047)	0.140	**0.027**
N to SI	0.181 (0.065)	0.172	**0.006**
E to SI	−0.278 (0.077)	−0.229	**<0.001**
A to SI	−0.068 (0.072)	−0.058	0.342
N to SI (indirect)	0.085	0.081	N/A
E to SI (indirect)	−0.099	−0.081	N/A
A to SI (indirect)	−0.025	−0.021	N/A

Note: Standard errors and *p*-values not available for indirect pathway estimates due to missing values. In bold, effects significant at the 0.05 threshold.

**Table 3:** Estimated unstandardized (with standard errors) and standardized parameters for the regression weights, as well as fit indices, for the two models with AC as mediator in the relationship between FFM variables and SI.

**Model 3, fit indices: *χ*^2^ = 142.311, df = 15, *p* < 0.001, CFI = 0.111, RMSEA = 0.206 90% CI (0.176–0.238)**			
Parameter estimates	Unstandardized *β*	Standardized *β*	*p*-value
N to AC	−0.069 (0.061)	−0.076	0.257
E to AC	−0.185 (0.071)	−0.174	**0.009**
O to AC	0.024 (0.068)	0.024	0.725
A to AC	−0.098 (0.069)	−0.095	0.155
C to AC	0.191 (0.051)	0.251	**<0.001**
AC to SI	0.306 (0.082)	0.257	**<0.001**
N to SI (indirect)	−0.021	−0.019	N/A
E to SI (indirect)	−0.057	−0.045	N/A
O to SI (indirect)	0.007	0.006	N/A
A to SI (indirect)	−0.030	−0.024	N/A
C to SI (indirect)	0.058	0.064	N/A
**Model 4, fit indices: *χ*^2^ = 92.562, df = 10, *p* < 0.001, CFI = 0.423, RMSEA = 0.203 90% CI (0.166–0.242)**			
Parameter estimates	Unstandardized *β*	Standardized *β*	*p*-value
N to AC	−0.068 (0.061)	−0.075	0.266
E to AC	−0.185 (0.071)	−0.175	**0.009**
O to AC	0.023 (0.068)	0.023	0.731
A to AC	−0.098 (0.069)	−0.096	0.153
C to AC	0.191 (0.051)	0.252	**<0.001**
AC to SI	0.293 (0.077)	0.247	**<0.001**
N to SI	0.349 (0.066)	0.322	**<0.001**
E to SI	−0.327 (0.078)	−0.260	**<0.001**
O to SI	−0.071 (0.074)	−0.059	0.336
A to SI	−0.100 (0.074)	−0.082	0.179
C to SI	0.034 (0.057)	0.038	0.548
N to SI (indirect)	−0.020	−0.018	N/A
E to SI (indirect)	−0.054	−0.043	N/A
O to SI (indirect)	0.007	0.006	N/A
A to SI (indirect)	−0.029	−0.024	N/A
C to SI (indirect)	0.056	0.062	N/A

Note: Standard errors and *p*-values not available for indirect pathway estimates due to missing values. In bold, effects significant at the 0.05 threshold.

**Table 4:** Estimated unstandardized (with standard errors) and standardized parameters for the regression weights, as well as fit indices, for a model with PB and TB (model 5) and a model with AC (model 6) as mediators in the relationship between FFM variables and SI.

**Model 5, fit indices: *χ*^2^ = 60.073, df = 7, *p* < 0.001, CFI = 0.681, RMSEA = 0.195 90% CI (0.151–0.242)**			
Parameter estimates	Unstandardized *β*	Standardized *β*	*p*-value
N to PB	0.442 (0.091)	0.321	**<0.001**
O to PB	−0.272 (0.101)	−0.177	**0.007**
E to TB	−0.428 (0.110)	−0.261	**<0.001**
N to TB	0.268 (0.095)	0.189	**0.005**
PB to SI	0.250 (0.048)	0.327	**<0.001**
TB to SI	0.110 (0.047)	0.148	**0.020**
N to SI	0.181 (0.068)	0.172	**0.008**
E to SI	−0.291 (0.068)	−0.238	**<0.001**
N to SI (indirect)	0.140	0.133	N/A
E to SI (indirect)	−0.047	−0.039	N/A
O to SI (indirect)	−0.068	−0.058	N/A
**Model 6, fit indices: *χ*^2^ = 31.452, df = 5, *p* < 0.001, CFI = 0.705, RMSEA = 0.163 90% CI (0.111–0.219)**			
Parameter estimates	Unstandardized *β*	Standardized *β*	*p*-value
E to AC	−0.181 (0.071)	−0.172	**0.011**
C to AC	0.180 (0.051)	0.238	**<0.001**
AC to SI	0.302 (0.074)	0.253	**<0.001**
N to SI	0.328 (0.066)	−0.150	**<0.001**
E to SI	−0.350 (0.078)	−0.278	**<0.001**
E to SI (indirect)	−0.055	−0.043	N/A
C to SI (indirect)	0.054	0.060	N/A

Note: Standard errors and *p*-values not available for indirect pathway estimates due to missing values. In bold, effects significant at the 0.05 threshold.
